# On the effect of COVID-19 pandemic in the excess of human mortality. The case of Brazil and Spain

**DOI:** 10.1371/journal.pone.0255909

**Published:** 2021-09-02

**Authors:** Jorge A. Borrego–Morell, Edmundo J. Huertas, Nuria Torrado

**Affiliations:** 1 Departamento de Matemática, UFRJ–Universidade Federal do Rio de Janeiro, Campus Santa Cruz da Serra, Duque de Caxias, Rio de Janeiro, Brazil; 2 Departamento de Física y Matemáticas, Universidad de Alcalá, Alcalá de Henares, Madrid, Spain; 3 Departamento de Análisis Económico: Economía Cuantitativa, Universidad Autónoma de Madrid, Madrid, Spain; Fuzhou University, CHINA

## Abstract

Excess of deaths is a technique used in epidemiology to assess the deaths caused by an unexpected event. For the present COVID–19 pandemic, we discuss the performance of some linear and nonlinear time series forecasting techniques widely used for modeling the actual pandemic and provide estimates for this metric from January 2020 to April 2021. We apply the results obtained to evaluate the evolution of the present pandemic in Brazil and Spain, which allows in particular to compare how well (or bad) these countries have managed the pandemic. For Brazil, our calculations refute the claim made by some officials that the present pandemic is “a little flu”. Some studies suggest that the virus could be lying dormant across the world before been detected for the first time. In that regard, our results show that there is no evidence of deaths by the virus in 2019.

## 1 Introduction

The first fatal reported cases of COVID–19 emerged as a mysterious pneumonia in late 2019 in Wuhan City, Hubei Province of China [1, 2]. Some recent studies suggest that the virus was already present across the world and simply could be lying inactive for years before been detected for the first time in China, [3]. The cause of the disease was confirmed as a new kind of highly contagious virus that is 96% identical at the whole-genome level of bat coronavirus samples, 79.6% identical to SARS–CoV and it is suspected of having a zoonotic origin [4, 5].

Although vaccination has started in a growing number of countries, Covid-19 continues to spread. At the present date, according to Our World in Data webpage (https://ourworldindata.org/covid-deaths), the highest number of infection cases have been reported in the United States, Brazil and the United Kingdom. The number of deaths is an obvious international comparator but ultimately an unreliable metric for direct comparisons. Moreover, the limited availability of viral testing and the imperfect sensitivity of the tests mean that the number of confirmed deaths may not be an accurate count of the true number of deaths caused by the virus [6–8]. For this reason, it is very important to have an effective planning of the health infrastructure and services based on accurate estimations given by mathematical models.

On the other hand, excess of deaths is a technique used in epidemiology which is typically applied to count the deaths caused by an event such as a heatwave or a pandemic that would not occur if the event would not happen. This methodology has been used to quantify the official undercounting of deaths for many pathogens, including pandemic influenza viruses and HIV [9–13], and it has been already studied by several authors to assess the impact of the present pandemic [14–17]. This metric avoids miscounting deaths from the under-reporting of COVID–19 related deaths and other health conditions left untreated and it is frequently used to measure the mortality impact of a crisis when not all causes of death are known. The excess deaths at lag *h*, *y*_*h*_, can be calculated by the formula

$$\begin{array}{c} y_{h}=O_{h}-U_{h}, \end{array}$$ (1)

where *O*_*h*_ is the observed number of deaths at lag *h* and *U*_*h*_ is the upper 95% or 80% prediction interval of the expected number of deaths at lag *h* under normal conditions.

At the present time, some academic institutions compile number of deaths from national databases of several countries and make available this information weekly, (see for instance, http://www.euromomo.eu and http://www.mortality.org). Unfortunately, an updated information of some countries is not available (particularly, as is the case of Brazil), and therefore, the excess of deaths could be underestimated. Thus, at least a model based in an accurate forecasting method would be of extreme importance.

Our objective is twofold. Firstly, we analyze whether there has really been an excess of deaths during the period from January 2020 to April 2021 in Brazil and Spain, by computing *y*_*h*_ defined as in (1) for each country. In order to obtain *U*_*h*_ (the upper 95% or 80% prediction interval of the expected number of deaths at lag *h*), we compare accurately three estimation methods and select the best performing technique to each country for the death counts, from January 2015 to December 2019, in the absence of the COVID-19 pandemic. Later, for these two best-performing models, one for each country, we obtain the expected death counts for the next 16 months starting in January 2020. The second purpose of this work is to compare how two nations of very different nature, population and idiosyncrasy, face the same external agent (COVID-19 disease), and what effects this same agent has on the excess mortality in both countries. To do this, we use the following percent relative

$$\begin{array}{c} P\equiv \text{Percent}\;\text{relative}=\frac{O_{h}-U_{h}}{U_{h}}100\%=\frac{y_{h}}{U_{h}}100\%, \end{array}$$ (2)

since it is well known that *y*_*h*_ is not an adequate measure to compare across countries.

In [17], it is studied the excess of mortality of Standardized Mortality Ratios for several Brazilian states. In the present manuscript we give an estimate for this metric for the whole Brazil. Apparently, Brazil is one of the countries with the highest number of deaths caused by the current pandemic. According to the Human Development Index or HDI (http://hdr.undp.org), which the United Nations elaborates to measure the progress of a country and which ultimately shows us the standard of living of its inhabitants, Brazil ranks 77th out of 189 countries. In terms of life expectancy, Brazil places in position 75 of 192 countries, specifically, their life expectancy is 76.57 years. On the other hand, Spain is in position 30 of the population table, made up of 196 countries and has a moderate population density. Spain ranks 25th out of 189 countries according to the HDI and 7th out of 192 countries with a life expectancy of 83.99 years. Clearly, demographic, population, social and economic characteristics of these two countries are very different and, in addition, the governments policies of these two countries against the pandemic have also been very different. In particular, the Brazilian government decided not to isolate its population entirely, while in Spain, its inhabitants were isolated for several months. The purpose of isolation and quarantine is to prevent the transmission of COVID-19 by restricting the movement and activities of people. Our results clearly show the positive effect of the isolation and other measures to halt the progression of the pandemic.

The rest of the manuscript is organized as follows. In Section 2 we show the data source and present the forecasting methods to be used. In Section 3 we discuss the best performing model, compute the excess of mortality and apply these results to provide some estimates of the deaths and discuss its significance in the actual pandemic. Finally, in Section 4, we present some concluding remarks.

## 2 Materials and methods

The Brazilian Institute of Geography and Statistics (IBGE) and the Spanish National Institute of Statistics (INE) are the main demographic data providers in Brazil and Spain, respectively, and offer an open and complete vision of countries data. Since the IBGE and INE institutions do not have still the complete information corresponding to 2020 and 2021, the observed total number of deaths for these two years were taken directly from civil registries (CNIRC for Brazil and MoMo for Spain), these institutions collect monthly and daily demographic data, respectively. In the Spanish case, MoMo’s daily data is one week late on the reporting date. The data used in this study were collected in mid-May 2021, to make sure that the total of deaths by any causes cannot be undercount the real death data. To our knowledge, our analysis makes use of the most recent available mortality data set with the highest possible completeness.

The Brazilian and Spanish data, from January 2015 to December 2019, were collected from the IBGE website http://www.ibge.gov.br and the INE website http://www.ine.es, respectively. Whereas the data, from January 2020 to April 2021, were collected from the CNIRC website https://transparencia.registrocivil.org.br/registros and from MoMo website http://www.isciii.es for Brasil and Spain, respectively. The data corresponding to the observed total number of deaths by any causes for both countries are available in the S1 Table.

**Fig 1:**
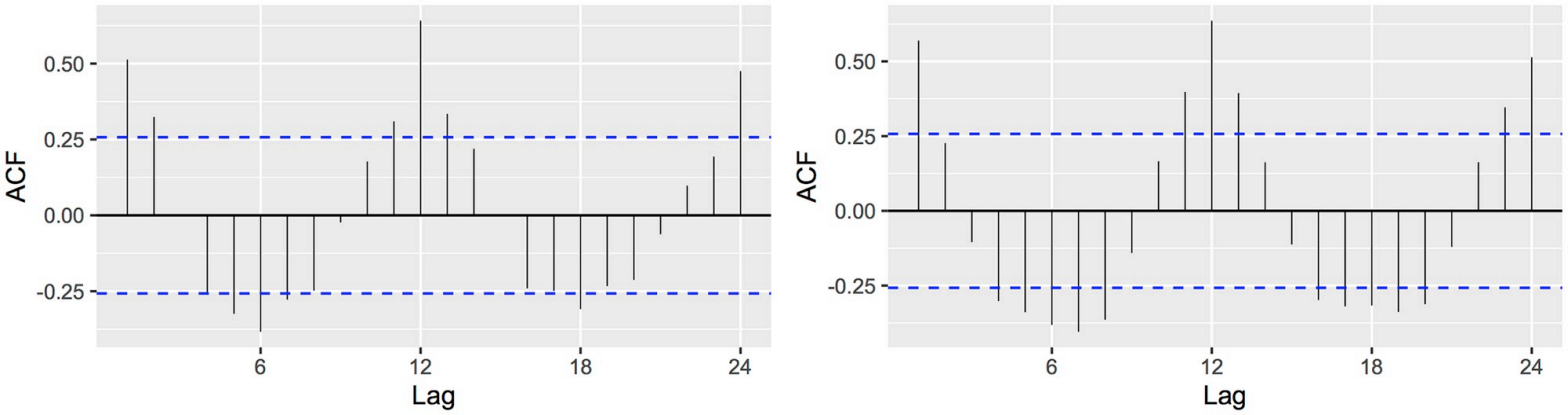
Correlograms of the series of total deaths from January 2015 to December 2019 of Brazil and Spain. Left hand side corresponds to Brazil and the right hand side corresponds to Spain. Lag units in months.

Some national statistical agencies (e.g. the United Kingdom Office for National Statistics (ONS) https://www.ons.gov.uk) use the difference in the average deaths from the past 5 years from the current total as a measure for calculating excess of deaths. This method could be appropriate for data without trend or seasonality. Usually, demographic time series have serial correlations. In particular, the correlograms in Fig 1 show a seasonal pattern of 12 months which means that the historical death data for both countries present both temporal trends and seasonality, consequently the average method in this case is not appropriate. We study instead the performance of three types of widely used models from univariate time series forecasting: the generalized linear model (GLM), the Autoregressive Integrated Moving Average (ARIMA), and the Exponential Smoothing (ETS). As a consequence, we obtain accurate estimates of the expected number of deaths by all causes from January 2020 to April 2021 based on monthly historical data from January 2015 to December 2019, that is, in the absence of the COVID-19 pandemic. Additionally, we calculate the excess of mortality and the percent relative through the definitions given in (1) and (2), respectively, for the data from January 2020 to April 2021, where *O*_*h*_ is the observed monthly total of deaths by all causes and *U*_*h*_ is the upper 95% prediction interval of the expected deaths for that period. All the computations were performed using the open-source R language version 4.0.4 (2020–06-22), operating system macOS Sierra 10.12.6, system x86_64, darwin 17.0.

Time series models have been widely used in economics, environmental sciences, and many other fields. These tools can be used for making short and long–term accurate estimates of the expected number of deaths based on the data. In particular, count time series usually appear in the demographic literature and other areas whenever a number of events per time period are observed and are usually modeled using several approaches, [18, 19]. In some cases, the sample space is large and it makes sense to approximate them by continuous random variables. Consequently, it seems plausible to expect that the existing theory of continuous random variables constitutes a good approximation. In that regard, ARIMA models have proven to be an effective forecasting tool when forecasting count time series associated with morbidity data cf. [20, 21]. We give now a background on the methods that we considered.

### 2.1 Generalized linear models

Generalized linear models (GLM) were originally introduced in [22], and provide under some conditions an unified theory suitable for continuous, categorical, and count data. The theory for these models was initially intended for independent data, however, under various assumptions it can be extended to dependent data. We refer to [18] and [19] for the general theory of GLM for time series.

Let $\left\{y_{t}:t\in N\right\}$ be a count time series and $\left\{x_{t}:t\in N\right\}$ a time-varying *r*–dimensional covariate vector or explanatory variables, say *y*_*t*_ = (*y*_*t*,1_, …, *y*_*t*,*r*_)′. Denote by $F_{t-1}$ the *σ*–field generated by *y*_*t*−1_, *y*_*t*−2_, …, *x*_*t*_, *x*_*t*−1_, …, i.e.,

$$\begin{array}{c} F_{t-1}=\sigma \left\{y_{t-1},y_{t-2},\ldots ,x_{t},x_{t-1},\cdots \right\}. \end{array}$$

Let

$$\begin{array}{c} \lambda_{t}=E\left[y_{t}|F_{t-1}\right], \end{array}$$

be the conditional mean of the response given the past with respect to some assumed distribution. Generalized linear models are defined as those types of models that solve the problem of relating λ_*t*_ to the covariates.

The ‘tscount’ package [23] implemented in R allows a general dependence structure for count time series and captures suitably the serial correlation among observations. Through the function tsglm(), the package fits by quasi conditional maximum likelihood–based estimation models in the general form

$$\begin{array}{c} g\left(\lambda_{t}\right)=\beta_{0}+\sum_{k=1}^{m}\beta_{k}\tilde{g}\left(y_{t-i_{k}}\right)+\sum_{\ell =1}^{n}\alpha_{l}g\left(\lambda_{t-j_{\ell }}\right)+\eta^{\prime }x_{t}, \end{array}$$

where $g:R^{+}\to R$ is a link function, $\tilde{g}:N\cup \left\{0\right\}\to R$ is a transformation function and ***η*** = (*η*_1_, …, *η*_*r*_)′ corresponds to the effects of covariates, which in our case are not present. The parameters *m* and *n* denote the number of lag observations and the lagged conditional means included in the model, respectively.

The assumption that the number of deaths is related to a Poisson distribution is found widely in the literature (see [24, Sect. 10.4] and the references therein). A classical example of this distribution in this context, involves the number of deaths caused by horse kicks to men in the Prussian Army between 1875 and 1894. In the present manuscript, we consider the Poisson and negative binomial distributions as the underlying distributions and compare its predictive power with others models.

### 2.2 Auto-regressive integrated moving average

The ARIMA model, introduced by Box–Jenkins in 1970s [25], is one of the most classic methods of time series analysis. It has been widely used to forecast infectious diseases. We refer to [26, 27] for background. The method is based on fitting an autoregression model of order *p* (AR(p)) combined with a moving average model of order *q* (MA(q)) defining an ARMA(p,q) model

$$\begin{array}{c} y_{t}=\mu +\phi_{1}y_{t-1}+\cdots +\phi_{p}y_{t-p}+\theta_{1}\epsilon_{t-1}+\cdots +\theta_{q}\epsilon_{t-q}+\epsilon_{t}, \end{array}$$

where *ϵ*_*t*_ is a random variable with mean zero and variance *σ*^2^. If the time series is not stationary in the mean, as is often the case in demography and epidemiology, a differencing transformation of order *d* will be applied to attain stationarity.

A non–seasonal ARIMA model is generally referred to as ARIMA(p,d,q) where *p* is the order of autoregression, *d* is the degree of differencing, and *q* is the order of moving average. In terms of the backward shift operator $B^{j}y_{t}=y_{t-j},j\in Z$, the model can be expressed symbolically as

$$\begin{array}{c} \phi \left(B\right)\left(1-B^{d}\right)(y_{t}-\mu \frac{t^{d}}{d!})=\theta \left(B\right)\epsilon_{t}, \end{array}$$

where *ϕ*(*z*) = 1 − *ϕ*_1_ *z* − ⋯ − *ϕ*_*p*_ *z*^*p*^ and *θ*(*z*) = 1 + *θ*_1_ *z* + ⋯ + *θ*_*q*_ *z*^*q*^ and *ϵ*_*t*_ is a white noise with zero mean and variance *σ*^2^. When *d* = 1, there is a trend with slope *μ*, often called “drift”.

Seasonal ARIMA models, also known as SARIMA, are also capable of modelling a wide range of seasonal data, as in our case. A seasonal ARIMA model is formed by including additional seasonal terms in the ARIMA model

$$\begin{array}{c} \Phi \left(B^{m}\right)\phi \left(B\right){\left(1-B^{m}\right)}^{D}{\left(1-B\right)}^{d}(y_{t}-\mu \frac{t^{d}}{d!})=\Theta \left(B^{m}\right)\theta \left(B\right)\epsilon_{t}, \end{array}$$ (3)

where Φ(*z*) = 1 − Φ_1_ *z* − ⋯ − Φ_*P*_ *z*^*P*^, *Θ*(*z*) = 1+*Θ*_1_ *z* + ⋯ + *Θ*_*Q*_ *z*^*Q*^ and *m* is the seasonal frequency. These class of models are denoted generically by ARIMA(p,d,q)(P,D,Q)_*m*_.

#### 2.2.1 Criteria for ARIMA model selection

We use the most recent automated selection of ARIMA model, provided via the ‘forecast’ package [28] of the R software known as automatic ARIMA, it includes both seasonal and non–seasonal variants and is executed through the function auto.arima(). A detailed description of the algorithm can be found in [29, 27, Sect. 9.7]. The algorithm is summarized as follows.

1. For the degree of differencing (*d*), the algorithm implemented in R allows obtaining this parameter using three different unit root tests: the Kwiatkowski-Phillips-Schmidt-Shin (KPSS), the Augmented Dickey-Fuller (ADF) and the Phillips-Perron (PP) tests, which are all well known in the literature about ARIMA models (see, e.g., [26]). In this study, we considered these three methods.
2. Following the Box-Jenkins methodology, after differencing *d* times the time series, the method requires identifying the stationary ARMA structure, i.e., obtaining the parameters *p* and *q*. To do this, the algorithm implemented in R minimizes the Akaike Information Criterion (AIC). Among all the options available in the R software, we used the options approximation = FALSE in order to avoid approximations to speed up the search, because this does not guarantee to find the best model and, on the other hand, stepwise = FALSE which allows to search for every combination of models given by *p* and *q*.

### 2.3 Exponential smoothing

Exponential smoothing methods are a class of time series forecasting techniques for univariate data and represent a powerful forecasting tool. These methods consider that a prediction is a weighted sum of past observations and use an exponentially decreasing weight for past observations (see [30, 31]). Three extra letters in the front of the method notation is used, to distinguish the models with additive and multiplicative errors. Thus, the triplet (E,T,S) refers to the three components: error, trend and seasonality. For instance, the model ETS(A,N,A), has additive errors, no trend and additive seasonality. In component form it can be expressed as

$$\begin{array}{cc} y_{t} & =l_{t}+s_{t-m}+\epsilon_{t},\;\text{(measurement}\;\text{or}\;\text{observation}\;\text{equation}),\\ l_{t} & =l_{t-1}+\alpha \epsilon_{t},\;\text{(state}\;\text{or}\;\text{transition}\;\text{equation}),\\ s_{t} & =s_{t-m}+\gamma \epsilon_{t},\;\text{(seasonal}\;\text{equation}), \end{array}$$

where *m* is the seasonal parameter, *α*, *γ* numerical coefficients and {*ϵ*_*t*_} is a white noise series which represents what is new and unpredictable and it is referred to as the errors or innovations.

The ‘forecast’ package for the R software through the ets() function, provides an efficient automatic selection from a taxonomy of over 30 possible options of forecasting models based in error, trend and seasonal components, [27, 30]. The best model selection is based on the AIC, AIC_*c*_ (this criteria is an AIC with a correction for small sample sizes) or the Bayesian information criterion (BIC).

### 2.4 Forecasting accuracy

The accuracy of a model can be tested by comparing the actual values with the predicted values. Recall that the forecast “error” is the difference between an observed value and its forecast,

$$\begin{array}{c} e_{T+h}=y_{T+h}-{\hat{y}}_{T+h}, \end{array}$$

where {*y*_1_, …, *y*_*T*_} are the observations in the training data set and {*y*_*T*+1_, …, *y*_*T*+2_, …, *y*_*T*+*N*_} are the observations in the test data set. In a similar way, residuals are equal to the difference between the observations and the corresponding fitted values,

$$\begin{array}{c} e_{t}=y_{t}-{\hat{y}}_{t}. \end{array}$$

In this manuscript we used the following measures for the forecasting accuracy:

$$\begin{array}{ccc} \text{Mean}\;\text{error}:ME & = & \frac{1}{c(I)}\sum_{k\in I}e_{k},\\ \text{Mean}\;\text{absolute}\;\text{error}:MAE & = & \frac{1}{c(I)}\sum_{k\in I}\left|e_{k}\right|,\\ \text{Root}\;\text{mean}\;\text{squared}\;\text{error}:RMSE & = & \sqrt{\frac{1}{c(I)}\sum_{k\in I}e_{k}^{2}},\\ \text{Mean}\;\text{absolute}\;\text{percentage}\;\text{error}:MAPE & = & \frac{100\%}{c(I)}\sum_{k\in I}|\frac{e_{k}}{y_{k}}|,\\ \text{Mean}\;\text{percentage}\;\text{error}:MPE & = & \frac{100\%}{c(I)}\sum_{k\in I}\frac{e_{k}}{y_{k}},\\ \text{Theil}\prime \text{s}\;\text{U}\;\text{statistic}:(\text{Theil}\prime \text{s}\;\text{U}) & = & \frac{RMSE_{1}}{RMSE_{2}},\\ \text{Atutocorrelation}\;\text{at}\;\text{lag}\;\text{1}:ACF1 & = & \frac{\hat{\gamma }\left(1\right)}{\hat{\gamma }\left(0\right)}, \end{array}$$

where RMSE_1_ is the RMSE of the chosen model, RMSE_2_ is the RMSE of the naïve method defined by ${\hat{y}}_{T+h}=y_{T},h\geq 1$ (all forecasts are equal to the value of the last observation), a value less than 1 of this statistic indicates a better performance on the average of the model as compared to the naïve method. The term in the last measure

$$\begin{array}{c} \hat{\gamma }\left(h\right)=\frac{1}{c(I)}\sum_{k=1}^{c(I)-|h|}\left(e_{k+|h|}-ME\right)\left(e_{k}-ME\right),\;-c\left(I\right)<h<c\left(I\right) \end{array}$$

denotes the sample autocovariance function, here the index *I* in the sum symbol denotes the training or test test and *c*(*I*) its number of elements. We also employed the time series cross–validation technique described in [27, Sect. 5.9], implemented in the tsCV() function of the ‘forecast’ R package to asses the forecast accuracy of the models.

## 3 Results and discussion

We compare now the performance of the three models over the datasets based on in-sample and out-of-sample procedures for each country, see [27, Sect. 9.10] and [32] for a background on the subject. In S1–S3 Files, we provide the results of the statistical analysis for the three models: GLM, ARIMA and ETS, respectively.

Firstly, it should be noticed that, by virtue of [27], the model ETS(A,N,A) for the Brazilian data described in S3 File can be expressed as an ARIMA(0,1,12)(0,1,0)_12_ model. However, this ARIMA model has more parameters than the ARIMA(1,0,1)(1,1,0)_12_ model given in S2 File. Hence, according to the parsimony principle (models with smallest possible number of parameters are preferable, [25]), we can expect a better performance of the ARIMA model given in S2 File than the ETS model given in S3 File for the Brazilian data.

In Tables 1 and 2, we report accuracy measures of the residuals for every models using the data from January 2015 to December 2019 as the training set, for Brazil and Spain, respectively. Note that the error measures of the three models almost do not differ, except the ETS in the Spanish dataset for the mean error giving as result 5.06 indicating a fit with almost no bias. However, a good behavior of the error measurements on the training set does not imply a good behavior on the test set.

**Table 1:** Accuracy measures for the models over the Brazilian data from January 2015 to December 2019.

	ME	RMSE	MAE	MPE	MAPE	ACF1
ARIMA	124.71	2245.08	1380.96	0.08%	1.31%	-0.02
ETS	103.51	2061.54	1546.25	0.05%	1.48%	0.03
ALNB	372.90	4528.23	3400.79	0.20%	3.26%	0.05

**Table 2:** Accuracy measures for the models over the Spanish data from January 2015 to December 2019.

	ME	RMSE	MAE	MPE	MAPE	ACF1
ARIMA	-44.85	1847.12	1120.43	-0.20%	3.03%	-0.04
ETS	5.06	1618.09	1051.22	-0.18%	2.81%	0.42
ALNB	20.32	2816.22	1849.28	-0.51%	4.99%	0.25

Next, by using a single forecasting origin, we split the initial data using 80% as training set and 20% as the test set. The accuracy measures are reported in Tables 3 and 4 for Brazil and Spain, respectively. Observe that the Theil’s U statistic is less than 1 for all cases except for the ALNB model in the Spanish case. When *U* < 1, the forecast is better than the naïve method and, if *U* > 1 then the forecast is worse than the naïve method. In addition, these tables suggest a better performance over the test set (corresponding to a forecast of 10 lags) by the ARIMA and ETS models for both countries.

**Table 3:** Accuracy measures of the models based on a split in training set (January 2015 to February 2019) and test sets (March 2019 to December 2019) for the Brazilian data.

	ME	RMSE	MAE	MPE	MAPE	ACF1	Theil’s U
Training set (ARIMA)	568.54	2525.20	1532.22	0.50%	1.45%	-0.002	0.49
Test set (ARIMA)	2094.77	2465.82	2302.43	1.86%	2.05%	0.34	
Training set (ETS)	81.32	2172.97	1610.00	0.03%	1.55%	0.02	0.58
Test set (ETS)	2148.03	3005.03	2667.15	1.89%	2.37%	0.53	
Training set (ALNB)	806.72	4111.25	2883.05	0.60%	2.78%	0.12	0.67
Test set (ALNB)	3307.83	3567.81	3307.83	2.93%	2.93%	-0.29	

**Table 4:** Accuracy measures of the models based on a split in training set (January 2015 to February 2019) and test sets (March 2019 to December 2019) for the Spanish data.

	ME	RMSE	MAE	MPE	MAPE	ACF1	Theil’s U
Training set (ARIMA)	5.92	1907.92	1153.94	-0.08%	3.08%	-0.02	0.65
Test set (ARIMA)	-261.03	1271.11	797.91	-0.74%	2.33%	-0.18	
Training set (ETS)	20.76	1737.65	1178.08	-0.11%	3.15%	0.45	0.49
Test set (ETS)	-173.02	1010.51	684.69	-0.44%	1.97%	-0.20	
Training set (ALNB)	-81.88	3933.45	2788.56	-1.06%	7.68%	0.08	1.34
Test set (ALNB)	-1210.22	2803.56	2349.11	-3.73%	6.91%	0.31	

**Fig 2:**
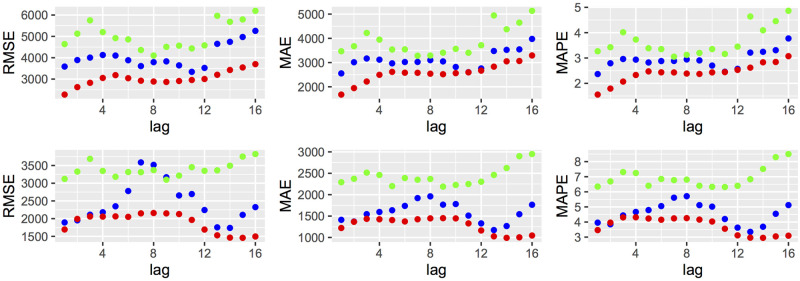
Accuracy measures from cross–validation. First row: Brazil; second row: Spain; ALNB(green), ARIMA(red) and ETS(blue).

On the other hand, Fig 2 displays the accuracy measures RSME, MAE and MAPE for the three models to each country by applying the cross-validation technique (initial parameter = 12) based on rolling-origin evaluations with re-estimation of the parameters and keeping the order of the model fixed at each step; [28] and [27, Sec. 5.10], using 16 steps forward. The ARIMA model for both countries shows a better performance for all lags. For this reason, we choose the ARIMA models as the best performing models.

**Fig 3:**
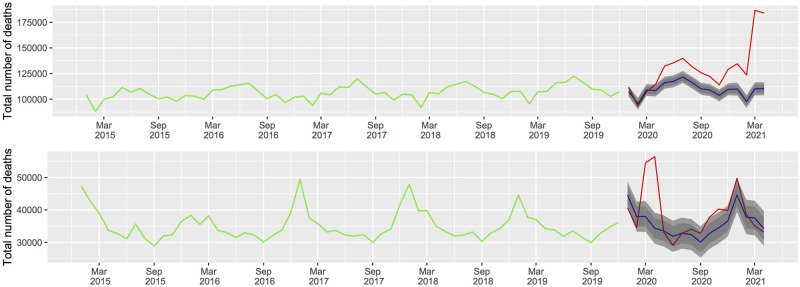
Total number of deaths by all causes observed and forecast of the ARIMA models. Left hand side Brazil; right hand side Spain; the green line corresponds to the observed deaths from January 2015 to December 2019; the red line corresponds to the observed deaths from January 2020 to April 2021; the blue line corresponds to the forecasts in the absence of the COVID-19 pandemic based on the ARIMA models with their respective prediction intervals. Lags units in months.

We obtain the expected death counts for the next 16 months starting in January 2020 in the absence of the COVID-19 pandemic for the two ARIMA models, one for each country. To do this, we use the forecast() function which belongs to the ‘forecast’ R package with a bootstrap strategy [28]. The expected death data are available in the S2 Table, and in Fig 3 we plot the observed deaths over the expected deaths for every month of reported data.

As can be appreciated from Fig 3, the observed number of deaths in January and February 2020 are very similar to their predictions for both countries. This means that there are not evidence of deaths by the virus before these months. However, there is a remarkable difference between the observed data and its point forecast from May 2020 to April 2021 for Brazil indicating three waves. On the other hand, for the Spanish case, it can be appreciated a first big wave during March-April 2020, a small second wave during October-November 2020 and a third wave on January 2021. This means that the number of deaths in those months has been higher than expected. Comparing the summer months of 2020 in Spain, which correspond to the holiday period, it can be seen that there is hardly any difference in July and August. Nevertheless, in June, the raw death counts attains the global minimum in the pandemic period which almost coincides with the lower 80% prediction interval. This may be due to mobility restrictions during the pandemic, which might lead to fewer deaths from road accidents.

Excess mortality can be defined as the expression given in (1). However, this measure is less comparable across countries due to large differences in populations. Because of this, we utilize the measure defined in (2). This measure calculates excess mortality as the percentage difference between the number of monthly deaths and the upper 95% prediction interval in the same month. In Tables 5 and 6 we show the results for both countries. We find for Brazil in 2020 an excess of deaths of at least 8.56%,9.97%,9.70% and 7.90% relative to the upper 95% prediction interval in May, June, July, and August 2020, respectively, whereas for Spain we find 27.51% and 44.18% relative to March and April in 2020, respectively. As can be seen, in this first wave Spain suffered high levels of excess mortality, while Brazil experienced much more modest increases in mortality in these months. This is related to some steps undertaken in the first weeks of the pandemic. Perhaps another cause could be due to the fact that the life expectancy in Spain is higher than in Brazil. It is important to note that the two datasets combine all ages. Therefore, the percent relative of deaths is impacted by differences in mortality risk by age and countries’ age distributions. It is clear that countries with older populations will tend to have higher percent relative by default. An interesting future work will be to study the excess of mortality considering different age groups.

**Table 5:** Excess of deaths for the Brazilian dataset.

		Jan	Feb	Mar	Apr	May	Jun	Jul	Aug	Sep	Oct	Nov	Dec
2020	*y*_*h*_	-1094	-7786	-7569	429	10435	12295	12359	9658	9721	7334	4392	13712
	*P*	-0.97%	-7.69%	-6.60%	0.38%	8.56%	9.97%	9.70%	7.90%	8.38%	6.40%	4.01%	11.88%
2021	*y*_*h*_	18366	19495	69926	67539								
	*P*	15.82%	18.73%	60.02%	57.95%								

*y*_*h*_ excess of deaths at lag *h*; *P* percent relative to the upper 95% prediction interval. All the data were collected at the time of writing this manuscript.

**Table 6:** Excess of deaths for the Spanish dataset.

		Jan	Feb	Mar	Apr	May	Jun	Jul	Aug	Sep	Oct	Nov	Dec
2020	*y*_*h*_	-8026	-7941	11759	17289	-5132	-7545	-4833	-3150	-2068	195	1006	-1507
	*P*	-16.47%	-18.67%	27.51%	44.18%	-13.39%	-20.59%	-12.84%	-8.47%	-5.93%	0.52%	2.56%	-3.64%
2021	*y*_*h*_	-70	-4453	-7759	-6470								
	*P*	-0.14%	-10.33%	-18.11%	-16.33%								

*y*_*h*_ excess of deaths at lag *h*; *P* percent relative to the upper 95% prediction interval. All the data were collected at the time of writing this manuscript.

From Tables 5 and 6, it is evident that, at the very beginning, the effect of the pandemic was worse in Spain, one of the nations in Europe more affected by the COVID-19 virus during the first wave, than in Brazil. However, in the forthcoming months in Brazil, there is an increase in the number of deaths by any cause. Observe, from Fig 3(left), that the prediction intervals not contain the observed data (red line). A declaration made by the Brazilian president in April 2020 showed that the present COVID-19 pandemic was not so worrisome (see [33]). The data substantiate that the increasing number of total deaths in this country could be a direct consequence of the little effort in undertaking true national lockdowns, the lack of use of face-masks to contain the propagation of the virus, and the sluggish rollout of the vaccination, [34].

According to [35], on 31 January 2020 in Spain the virus started to spread, and on 14 March 2020, a lockdown was imposed. On 25 February 2020, the virus started to spread in Brazil [36], and 5 May 2020, São Luís, capital of Maranhão state, was the first city together with Fortaleza, capital of Ceará state, to enter the lockdown, [37]. On the other side, observe that in March 2020 the excess of deaths in Spain attained 27.51%, just matching up with the lockdown date. This measure had a positive effect, since in May 2020 the number of deaths decreased considerably, as the percent relative −13.39% confirms. A similar situation occurred in Brazil, as in May 2020 the excess of deaths was 8.56%, just when the isolation measures in some Brazilian capitals began. In this case, it is in August 2020 when the number of deaths decreases slightly. The state of alarm ended in June 2020 for Spain, and however a relaxation in the isolation measures brought a slight increase in the number of deaths in October and November 2020 which leads to reinforce again some mobility restrictions [38].

The very high peaks in Brazil in March and April 2021, 60.02% and 57.95% respectively, are probably a direct consequence of the sluggish rollout of the vaccination, which started in January 2021, [39]. According to the website https://ourworldindata.org/covid-vaccinations, on April 30, 2021 the percentage of the Brazilian population vaccinated was 13.71%, whereas on the same date was 25.15% for Spain, this and the above discussion explain the situation in the first months of 2021 for both countries showed in Fig 3 and Tables 5 and 6.

## 4 Concluding remarks

In the present manuscript, we give an estimate for the excess of mortality by all causes for Brazil and Spain. This metric is an appropriate international measure which avoids the undercounting of deaths by many pathogens, including the actual pandemic. Together with official deaths reported for COVID-19, the excess mortality provides also a tool in evaluating the effects of an ongoing pandemic.

Using monthly mortality data from 2015 to 2019, we perform a forecast for the expected number of total deaths for 2020 and the first four months of 2021 in the absence of the COVID-19 pandemic. To do this, we studied the accuracy of three different forecasting methods widely used in count data. We found that the class of ARIMA models give the best results as compared to ETS and GLM. We considered, within the GLM class, the autoregressive Poisson and the negative binomial models, together with a logarithmic transformation on the parameter, and we found in particular better performance of ARIMA and ETS models over those in the GLM class. It is known that mortality data generally show overdispersion and this can be the reason for the best performance of the other models, at least for the for the countries considered in this manuscript. Undoubtedly, the comparative assessment of these models for a similar study in past pandemics and over other countries is an interesting problem to address in the future.

Some studies in the literature [3] suggest that the COVID-19 virus could be lying dormant across the world before been detected for the first time. Notice that, in case of an uncommon excess of deaths in 2019, the error measures over the test sets reported in Fig 2 from the cross-validation technique would detect abnormal peaks, which does not hold. Furthermore, the 95% prediction intervals contain the observed number of deaths for both countries for the first two months of 2020. Hence, there is no evidence of deaths by the virus prior to these months in neither of the two countries.

When the mortality data are not available completely, it is possible to underestimate the excess of deaths, which means that this metric can be actually higher. One option might be to wait for the information in the databases to be up to date, and then, based on the techniques described in this manuscript, a complete description of the effect of the pandemic can be easily estimated. Another option would be to use methods to adjust death counts for incompleteness. These techniques allows to obtain appropriate correction of the observed data to estimate the number of death which have occurred, but not yet reported (see, e.g. [40]). In adition, it would be interesting to study the effect of the COVID-19 pandemic on the excess of deaths by age groups and sex to examine possible differences between the two countries studied in this work. We are currently working on these problems and hope to report the findings in a future paper.

## Supporting information

S1 Table Number of deaths by all causes for Brazil and Spain in the period from January 2015 to April 2021. (PDF)

S2 Table Forecast of the best performing ARIMA model. (PDF)

S1 File Analysis of the best performing GLM model. (PDF)

S2 File Analysis of the best performing ARIMA model. (PDF)

S3 File Analysis of the best performing ETS model. (PDF)
