# Supplementary material for: On the effect of COVID-19 pandemic in the excess of human mortality. The case of Brazil and Spain
Source: PLoS One. 2021 Sep 2;16(9):e0255909. doi: 10.1371/journal.pone.0255909 (PMC8412318; doi:10.1371/journal.pone.0255909)
Supplement: S1 Table — (PDF) [file pone.0255909.s001.pdf]

Table 1: **Number of total deaths by all causes in Brazil from 2015 to April 2021.**

	Jan	Feb	Mar	Apr	May	Jun	Jul	Aug	Sep	Oct	Nov	Dec
2015	104045	87956	99836	102191	111355	106899	110469	104752	100332	101913	98019	103633
2016	102884	99931	108711	109295	112709	114202	115376	108070	100379	104579	96607	101907
2017	102883	94061	105762	104145	111944	111401	119851	112116	104895	106524	99215	104782
2018	104098	91992	106467	105236	112180	114413	117129	112573	106568	104866	100527	107447
2019	107740	95468	107161	107595	115952	116373	122306	116104	109720	108788	102754	107331
2020	111500	93416	107096	114689	132304	135600	139826	131835	125725	121981	113942	129134
2021	134460	123530	186432	184095								

Source: 01–2015 to 12–2019 (IBGE) and 01–2020 to 04–2021 (CNIRC) (at the time of writing this manuscript).

Table 2: **Number of total deaths by all causes in Spain form 2015 to April 2021.**

	Jan	Feb	Mar	Apr	May	Jun	Jul	Aug	Sep	Oct	Nov	Dec
2015	47269	42464	38987	33686	32706	31078	35628	31220	28863	31898	32346	36423
2016	38334	35548	38198	33707	33001	31512	32961	32248	30095	32060	33759	39188
2017	49370	37434	35779	33208	33673	32236	31924	32356	30049	32680	34149	41665
2018	47911	39756	39772	34956	33421	31970	32215	33177	30293	32879	34338	37033
2019	44596	37711	37041	34181	33853	31858	33528	31633	29875	32672	34623	36054
2020	40699	34586	54498	56426	33184	29105	32799	34038	32802	37760	40276	39831
2021	49737	38666	35095	33147								

Source: 01–2015 to 12–2019 (INE) and 01–2020 to 04–2021 (MoMo) (at the time of writing this manuscript).
