# Supplementary material for: On the effect of COVID-19 pandemic in the excess of human mortality. The case of Brazil and Spain
Source: PLoS One. 2021 Sep 2;16(9):e0255909. doi: 10.1371/journal.pone.0255909 (PMC8412318; doi:10.1371/journal.pone.0255909)
Supplement: S2 Table — (PDF) [file pone.0255909.s002.pdf]

Table 1: **Forecast from the ARIMA model for Brazil.**

	Point.Forecast	Lower 80%	Lower 95%	Upper 80%	Upper 95%
2020 Jan	107513	104191	102432	110836	112594
2020 Feb	95544	91844	89885	99243	101202
2020 Mar	108877	105092	103089	112661	114665
2020 Apr	108441	104636	102622	112246	114260
2020 May	116042	112233	110216	119852	121869
2020 Jun	117477	113666	111649	121287	123305
2020 Jul	121638	117827	115809	125449	127467
2020 Aug	116348	112537	110520	120159	122177
2020 Sep	110175	106364	104347	113986	116004
2020 Oct	108819	105007	102990	112630	114647
2020 Nov	103721	99910	97893	107533	109550
2020 Dec	109593	105782	103765	113404	115422
2021 Jan	109837	105746	103580	113928	116094
2021 Feb	97700	93545	91345	101856	104055
2021 Mar	110127	105956	103748	114298	116506
2021 Apr	110172	105998	103788	114346	116556

Table 2: **Forecast from the ARIMA model for Spain.**

	Point.Forecast	Lower 80%	Lower 95%	Upper 80%	Upper 95%
2020 Jan	44591	41887	40456	47294	48725
2020 Feb	37894	34865	33261	40923	42527
2020 Mar	37987	34880	33235	41094	42739
2020 Apr	34355	31228	29573	37481	39137
2020 May	33526	30395	28737	36658	38316
2020 Jun	31859	28726	27068	34992	36650
2020 Jul	32840	29706	28048	35973	37632
2020 Aug	32395	29262	27603	35529	37188
2020 Sep	30078	26945	25286	33211	34870
2020 Oct	32772	29639	27980	35906	37565
2020 Nov	34478	31344	29686	37611	39270
2020 Dec	36545	33412	31753	39679	41338
2021 Jan	44593	41183	39378	48002	49807
2021 Feb	37802	34325	32485	41278	43119
2021 Mar	37511	34017	32168	41005	42854
2021 Apr	34267	30769	28918	37765	39617
