# Supplementary material for: On the effect of COVID-19 pandemic in the excess of human mortality. The case of Brazil and Spain
Source: PLoS One. 2021 Sep 2;16(9):e0255909. doi: 10.1371/journal.pone.0255909 (PMC8412318; doi:10.1371/journal.pone.0255909)
Supplement: S1 File — (PDF) [file pone.0255909.s003.pdf]

The best–performing GLM model

Taking into account the serial dependence, we performed a regression on previous observations using the tsglm() function. By applying scoring rules for the selection of the distributions (scoring() function of the package 'tscount') and using the probability integral transform histogram (pit() function of the package 'tscount'), summarized below, we obtained the best results by assuming the negative binomial as the conditional distribution and considering $g(x) = \log x$ and $\tilde{g}(x) = x$ as link and transformation functions respectively. We obtained the models

$$\log \lambda_t = 0.58 + 0.41 y_{t-1} + 0.77 y_{t-12} - 0.40 \lambda_{t-2} + 0.16 \lambda_{t-12}, \quad \text{Brazil}, \tag{1}$$
$$\log \lambda_t = 0.64 + 0.24 y_{t-1} + 0.45 y_{t-12} - 0.15 \lambda_{t-2} + 0.40 \lambda_{t-12}, \quad \text{Spain}, \tag{2}$$

the overdispersion coefficient 'sigmasq' seems to be small and was estimated as 0.002 for Brazil and 0.006 for Spain. The negative binomial assumption, $y_t \mid \mathcal{F}_{t-1} \sim \text{NegBin}(\lambda_t, \theta)$, for the conditional distribution implies that

$$P(y_t = y \mid \mathcal{F}_{t-1}) = \frac{\Gamma(\theta + y)}{\Gamma(y+1)\Gamma(\theta)} \left(\frac{\theta}{\theta + \lambda_t} \right)^{\theta} \left(\frac{\lambda_t}{\theta + \lambda_t} \right)^{\theta},$$

where λ_t satisfies (1) for Brazil and (2) for Spain and the corresponding dispersion parameter $\theta = \frac{1}{\text{sigmasq}} = 500$ for Brazil and 166.67 for Spain. We will refer in the present manuscript to (1) or (2) as the ALNB model (autoregressive log–negative binomial model).

The plot of the autocorrelation function of the residuals is given in Figure 1 and shows them inside the threshold limits represented by dashed lines in blue, indicating that the residuals are behaving like a white noise. In order to assess the probabilistic calibration of the predictive distribution, we plotted the probability integral transform (PIT), as described in [1, 2]. The PIT histogram is typically used informally as a diagnostic tool, deviations from uniformity hint forecast failures and model deficiencies. U–shapes histograms indicates underdispersion of the predictive distribution, whereas an upside down U–shape indicate overdispersion. By using the pit() function () of the 'tscount' R package, we found that the plot of the probability integral transform histogram, given in Figure 2 for both countries, show a slight upside down U–shape histograms, indicating overdispersion in the predictive distributions, hence we can expect forecast failures. In that regard, we found a better performance of ARIMA and ETS models based on the cross–validation technique, as discussed in [3, Sect. 5.9]. Poisson and binomial assumptions are found widely in the actuarial literature, as pointed out in [4, Sect. 10.4]. Mortality data generally show more variation than is allowed for these models so that, the assumption that mortality data follow one of these distributions is not correct. In particular, our findings also show that the actual underlying distribution for our data should not be Poisson nor negative binomial.

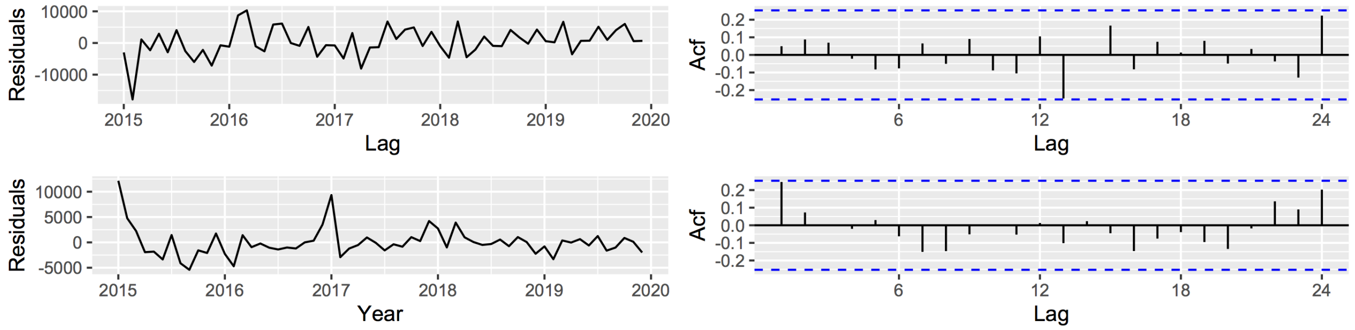

Figure 1: **Residuals from the ALNB model.** The first and second row represent the residuals and the correlation function for the ALNB Brazilian and Spanish models respectively. Lag units in months.

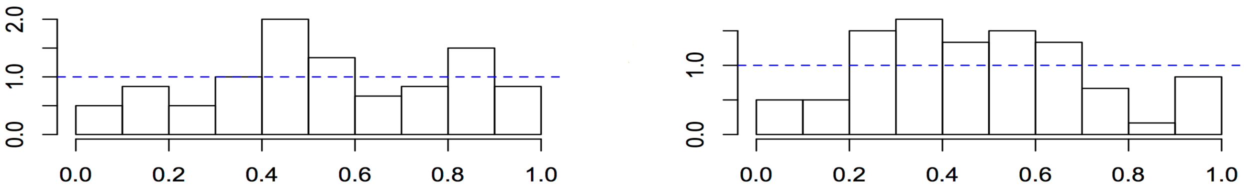

Figure 2: **Probability integral transform of the models.** Left hand side: Brazil; right hand side: Spain.

References

[1] Czado C, Gneiting T, Held L. Predictive Model Assessment for Count Data, Biometrics 2009 December; 65, 1254–1261.

[2] Gneiting T, Balabdaoui F, Raftery AE. Probabilistic Forecasts, Calibration and Sharpness. *Journal of the Royal Statistical Society B* 2007; 69(2), 243–268.

[3] Hyndman RJ, Athanasopoulos G. Forecasting: principles and practice, 3rd ed. OTexts: Melbourne, Australia; 2019. Avaible at: `https://otexts.com/fpp3/`.

[4] Macdonald AS, Richards SJ and Currie ID. Modelling mortality with actuarial applications. International Series on Actuarial Science, Cambridge University Press; 2018.

[5] Nelder JA, Wedderburn RWM. Generalized linear models. *J. R. Stat. Soc. Ser. A-Stat. Soc.* 1972; *135*, 370–384.
