# Supplementary material for: On the effect of COVID-19 pandemic in the excess of human mortality. The case of Brazil and Spain
Source: PLoS One. 2021 Sep 2;16(9):e0255909. doi: 10.1371/journal.pone.0255909 (PMC8412318; doi:10.1371/journal.pone.0255909)
Supplement: S2 File — (PDF) [file pone.0255909.s004.pdf]

The best–performing ARIMA model

By applying the auto.arima() function of the forecast package [1] to the time series defined by the Brazilian dataset, the optimal model is selected as a seasonal ARIMA$(1,0,1)(1,1,0)_{12}$ with log likelihood=-446.17, AIC=900.34, AICc=901.27, BIC=907.82, and for the Spanish data the optimal model is selected as a seasonal ARIMA$(1,0,0)(1,1,0)_{12}$ with log likelihood=-436.39, AIC=878.77, AICc=879.32, BIC=884.39. The optimal models are:

$$(1+0.55B^{12})(1-0.49B)(1-B^{12})(y_t-118)=\epsilon_t, \quad \text{(Brazil)}, \tag{1}$$

$$(1+0.50B^{12})(1-0.50B)(1-B^{12})y_t=\epsilon_t, \quad \text{(Spain)}, \tag{2}$$

where $\hat{\sigma}^2 = 6720493$ (Brazil) and $\hat{\sigma}^2 = 4450232$ (Spain) are the estimated variances.

The autocorrelation function of the residuals is showed in Figure 1. Observe that for both countries, the residuals lie between the threshold limits defined by the dashed line in blue, indicating that the residuals are behaving like white noise. We also applied the Ljung-Box test for group autocorrelations of the time series is obtained by using the checkresiduals() function of the 'forecast' package, with automatic selection of the parameters. We obtain a p-value = 0.11 for Brazil and for the Spanish model we obtain a p-value =0.54, which are both significantly large. Hence, it is reasonable to assume that residual error series follows a white noise process (in the sense of a sequence of serially uncorrelated random variables with zero mean and finite variance).

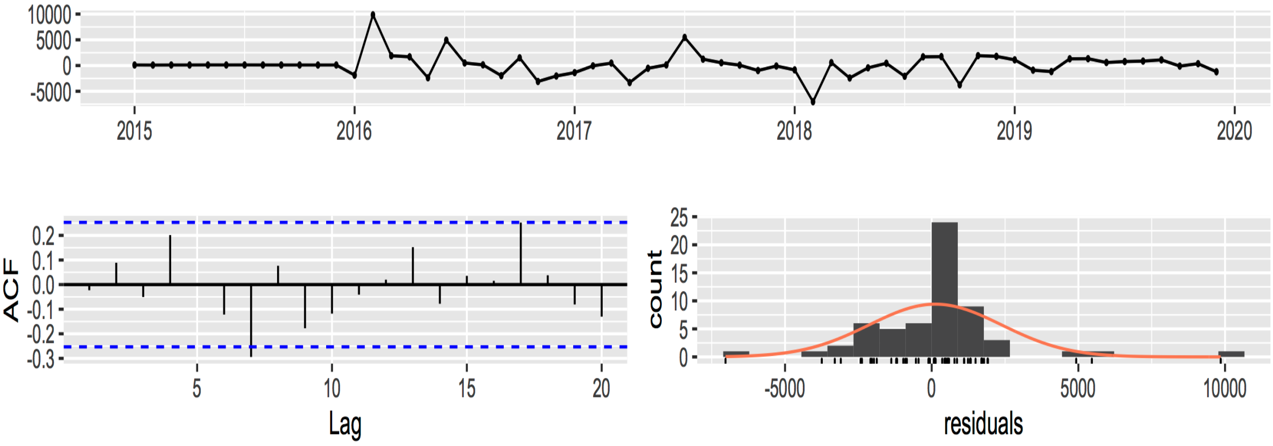

(a) Residuals from ARIMA$(1,0,1)(1,1,0)_{12}$ model for the Brazilian data.

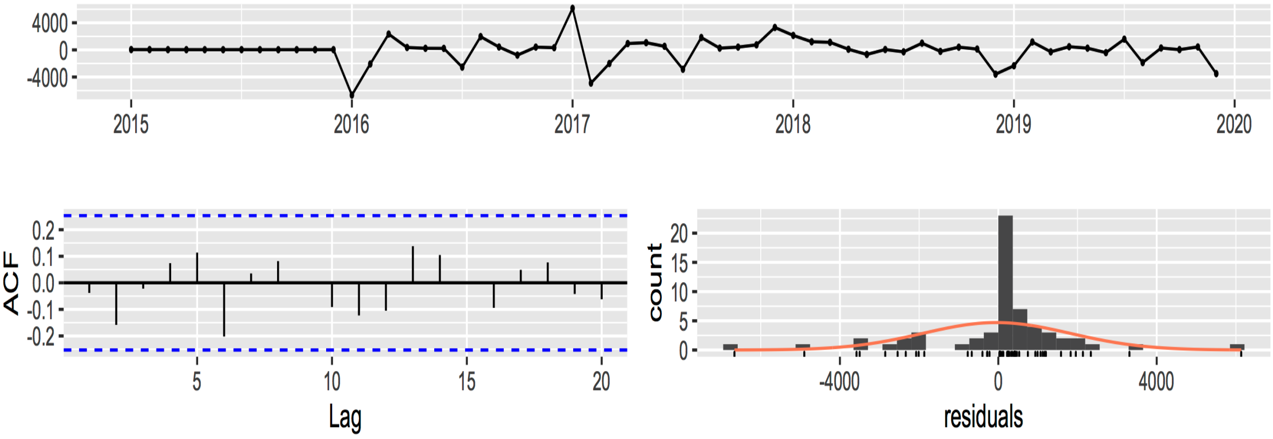

(b) Residuals from ARIMA$(1,0,0)(1,1,0)_{12}$ model for the Spanish data.

Figure 1: Residuals for the ARIMA models. The first row represents the residuals; the second row represents the autocorrelation and the histogram of the residuals.

References

[1] Hyndman RJ, Athanasopoulos G, Bergmeir C, Caceres G, Chhay L, O'Hara–Wild M, Petropoulos F, Razbash S (2020, March 31). Package "forecast", Version 8.12. https://cran.r-project.org/web/packages/forecast/forecast.pdf.
