# Supplementary material for: On the effect of COVID-19 pandemic in the excess of human mortality. The case of Brazil and Spain
Source: PLoS One. 2021 Sep 2;16(9):e0255909. doi: 10.1371/journal.pone.0255909 (PMC8412318; doi:10.1371/journal.pone.0255909)
Supplement: S3 File — (PDF) [file pone.0255909.s005.pdf]

The best–performing ETS model

The application of the ets() function of the forecast package [1] to the data of the IBGE from January 2015 to December 2019, produces an ETS(A,N,A) model with AIC=1191.406, AICc= 1202.315 and BIC= 1222.821. The measurement and transition equations obtained of the state space model have the form:

$$\begin{aligned} y_t &= l_t + s_{t-12} + \epsilon_t, \\ l_t &= l_{t-1} + 0.64\epsilon_t, \\ s_t &= s_{t-12} + 10^{-4}\epsilon_t, \end{aligned} \tag{1}$$

where

$$\begin{aligned} l_0 &= 105487.69, \\ (s_0, \ldots, s_{-12}) &= (-1696.62, -7145.66, -1314.64, -2802.18, 3629.00, 10137.31, \\ &\quad 6827.75, 6432.20, -414.95, 123.49, -11338.13, -2437.56) \end{aligned}$$

and the innovations, ϵ_t, should represent a white noise with mean 0 and constant variance. The estimated variance in this model is $\hat{\sigma} = 2354.44$.

In the Spanish case, the optimal ETS model is an ETS(M,N,M) which has multiplicative errors, no trend and multiplicative seasonality, with AIC=1145.25, AIC$_c$=1156.16 and BIC=1176.66. The measurement and transition equations have the form:

$$\begin{aligned} y_t &= l_{t-1}s_{t-12}(1 + \epsilon_t), \\ l_t &= l_{t-1}(1 + 2 \cdot 10^{-04}\epsilon_t), \\ s_t &= s_{t-12}(1 + 10^{-04}\epsilon_t). \end{aligned} \tag{2}$$

where

$$\begin{aligned} l_0 &= 35045.98, \\ (s_0, \ldots, s_{-12}) &= (1.09, 0.97, 0.93, 0.85, 0.92, 0.95, 0.91, 0.95, 0.97, 1.08, 1.09, 1.29) \end{aligned}$$

and the errors or innovations ϵ_t should represent a white noise. The estimated variance of the errors is $\hat{\sigma} = 0.05$.

The plots of the errors and its autocorrelation function are given in Figure 1. Notice that some values are slightly out of the threshold limits defined by the dashed line in blue for the Brazilian model. The Ljung-Box test with automatic selection of the parameters for group autocorrelation of the errors gives the p-value $= 3.61 \cdot 10^{-4}$ for the Brazilian model and a p-value $= 5.66 \cdot 10^{-07}$ for the Spanish model, both are significantly small. Hence, the assumption that the error series follows a white noise process is not satisfied, from where we conclude that there is still some hidden information that the models do not capture.

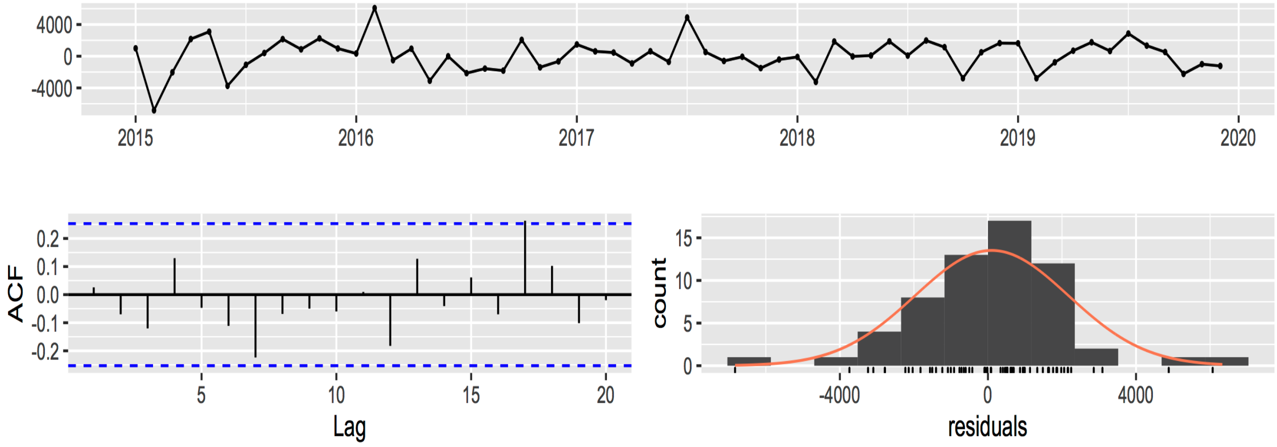

(a) Errors from ETS(A,N,A) model for the Brazilian data.

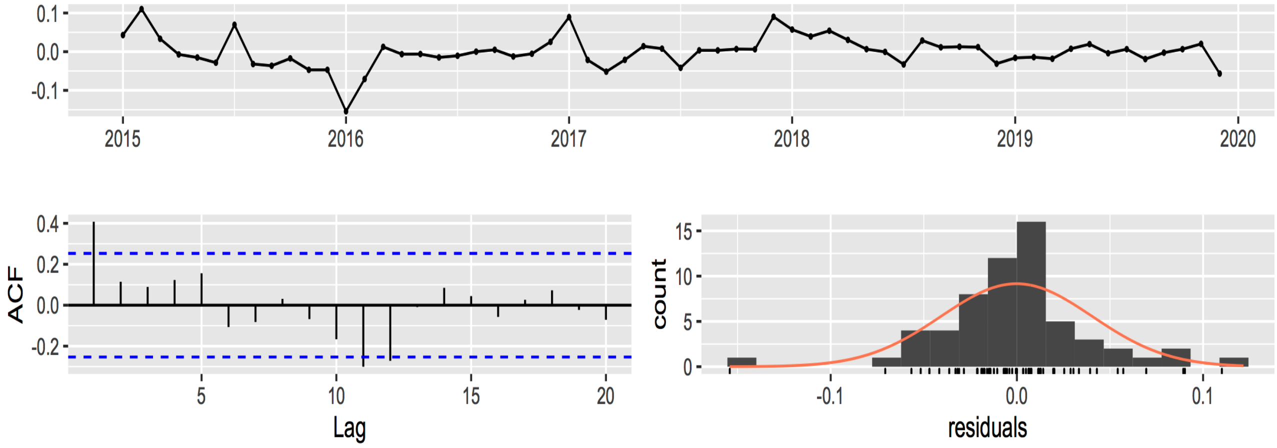

(b) Errors from ETS(M,N,M) model for the Spanish data.

Figure 1: For both figures, the first row represents the errors, the second row at the left represents the autocorrelation function of the residuals and the right represents the histogram of the residuals.

References

[1] Hyndman RJ, Athanasopoulos G, Bergmeir C, Caceres G, Chhay L, O'Hara–Wild M, Petropoulos F, Razbash S (2020, March 31). Package "forecast", Version 8.12. `https://cran.r-project.org/web/packages/forecast/forecast.pdf`.
